# From Intelligent Control to Integrated Autonomy: Reflections on Advanced Intelligent Robotics and Autonomous Control Systems

**DOI:** 10.3390/s26206460

**Published:** 2026-10-13

**Authors:** Tamás Haidegger, Levente A. Kovács, Péter Galambos

**Affiliations:** 1 University Research and Innovation Center (EKIK), Obuda University, 1034 Budapest, Hungary; 2 School of Computing, Queen’s University, Kingston, ON K7L 3N6, Canada; 3 Physiological Controls Research Center, University Research and Innovation Center (EKIK), Obuda University, 1034 Budapest, Hungary; 4 John von Neumann Faculty of Informatics, Obuda University, 1034 Budapest, Hungary; 5 Antal Bejczy Center for Intelligent Robotics, University Research and Innovation Center (EKIK), Obuda University, 1034 Budapest, Hungary

**Keywords:** intelligent robotics, embodied AI, physical AI, integrated autonomy, computational intelligence, cyber–physical systems, sustainable robotics, digital twins, robotic safety and validation

## Abstract

Robotics is advancing toward embodied intelligence and high-level autonomy, requiring the seamless integration of sensing, computation, communication, control, and human interaction to be designed as a coherent system. This Editorial synthesizes ten contributions to this Special Issue, entitled “Advanced Intelligent Robotics and Autonomous Control Systems—Celebrating the 75th Birthday of Professor Imre J. Rudas”, and relates their findings to computational intelligence and autonomous systems. Spanning robot navigation, human–robot safety, laboratory automation, digital pathology, manufacturing, medical robotics, and archeology, the collection highlights the complementary roles of hybrid reasoning, uncertainty management, network-aware control, digital twins, and reproducible sensor validation. Emerging research priorities include integrating learning methods with physical models and explicit constraints, coordinating distributed computation and control, and extending verification from individual components to system behavior. Multimodal AI offers further opportunities for task interpretation and adaptation, while reinforcing the need for auditable execution and deterministic safety mechanisms. The central perspective is that reliable autonomy depends on system integration and verifiable behavior alongside algorithmic performance.

## 1. Introduction

We are witnessing an accelerating change in engineering due to the development and advancement of intelligent methods and Artificial Intelligence (AI). This Special Issue “Advanced Intelligent Robotics and Autonomous Control Systems—Celebrating the 75th Birthday of Professor Imre J. Rudas” was conceived both as a scientific collection and as a reflection on a research trajectory that has helped shape modern intelligent engineering. Over several decades, Professor Rudas has contributed to the science of robot control, fuzzy systems, computational intelligence, information aggregation and—more recently—the theory of autonomous and complex systems. A recurring theme in this work is that useful intelligence emerges not from a single algorithmic paradigm, but from the purposeful integration of models, data, uncertainty handling, computation, and system-level feedback. His early works on combining artificial neural networks and fuzzy methods for robot control [1], as well as on fuzzy adaptation for nonlinear robotic systems [2], have already anticipated the hybridization process that is now becoming a cornerstone of autonomy concepts. Later contributions on information aggregation [3], autonomous systems [4], and systems of systems [5] broadened this view from individual control loops to increasingly interconnected and distributed intelligent machines and systems.

The ten papers published in this Special Issue provide a fair reflection of that transition. Their topics range from fuzzy signatures for robot path planning [6] and deep-learning-based human–robot safety [7] to autonomous laboratory workflows [8], network-aware robotic communication [9], cyber–physical precision pathology [10], digital twins for production systems [11], reproducible sensor validation [12], augmented-reality-assisted medical robotics [13], nonlinear mobile-robot control [14], and the use of robotics in archeology [15]. Altogether, these suggest that the next era of robotics is defined less by isolated improvements in perception or control, but by the integration of sensing, inference, communication, action, and validation into complete operational systems.

## 2. Recent Trends in Intelligent Robotics

One clear theme is the continued relevance of structured uncertainty and hybrid reasoning. Karadeniz et al. [6] examine how aggregation operators influence fuzzy signatures in robot path planning. It reflects a need to compress heterogeneous, uncertain observations into decision structures that remain interpretable and computationally manageable. The connection to Rudas’s work on aggregation functions in intelligent systems is direct [3]. Such methods retain value in current times, because robotic autonomy must often make a decision based on incomplete, noisy, or context-dependent information. We can therefore witness not a replacement of model-based and fuzzy techniques by general learning methods, but an adaptive combination of them.

A second theme is safety: system safety, calibration, and validation are becoming priority design objectives of human-centered technologies. Bricher and Müller [7] show how deep learning can be incorporated into an ISO/TS 15066-compliant human–robot safety framework to distinguish human body regions and adapt robot motion, reducing cycle time while respecting biomechanical constraints [16]. The standardization of emerging robot technologies remains a hot topic [17]. Kuti et al. [12], from a different direction, describe a robot arm as a high-precision reference for the systematic validation of orientation sensors. Sayadi et al. [13] integrate tele-kinesthesia, force feedback, and augmented reality for epidural interventions, placing perception and human guidance inside a safety-critical robotic loop. These studies indicate a broader shift: performance alone is no longer a sufficient measure of intelligence. Autonomous systems must also demonstrate traceability, repeatability, bounded behavior, and understandable failure modes.

Recent results also highlight the increasing importance of distributed cyber–physical system-level operation. Fernández-Madrigal et al. [9] address the statistical modeling of round-trip times for networked robot sensor transmissions, while Simion et al. [11] demonstrate cloud/VPN-based remote control and a digital twin architecture for a modular production system assisted by a mobile cyber–physical robot. Communication delay, computational placement, and synchronization are therefore not peripheral engineering details; these directly shape closed-loop behavior. This observation is echoed by recent work involving Rudas on guaranteed-performance robotic manipulation under limited communication resources [16]. As intelligence is partitioned among robots, edge devices, local servers, and cloud services, the communication–computation–control continuum will increasingly need to be designed as a single system.

Other contributions show that autonomy is moving from robot-centric tasks toward end-to-end domain workflows. Kucarov et al. [8] map manual laboratory operations to robot movements to create an autonomous digital pathology workflow, while a companion study integrates morphology, morphometry, and genomic information in a cyber–physical precision-oncology pipeline [10]. Kyriakoulia et al. [15] survey robotics in archeology, where sensing, mobility, 3D reconstruction, and remote operation can extend human access to fragile or inaccessible environments. Together with the compensation-based mobile-robot control of Shadrin et al. [14], these papers demonstrate how autonomy is increasingly evaluated in the context of a complete mission rather than a laboratory benchmark.

## 3. Next Generation of Intelligent Robotics

The trajectory of research traceable across this Special Issue points toward several likely developments. First, hybrid intelligence will become a practical necessity. General-purpose learning models will continue to expand the semantic capabilities of robots, but safety-critical autonomy will require them to operate inside architectures that include physical models, constraints, uncertainty estimates, and explicit supervisory logic. There exists a research line from fuzzy adaptation and information aggregation [1,2,3] to contemporary data-driven robotics.

Second, cloud and network awareness will become intrinsic to autonomy. In distributed robotic systems, latency, packet loss, bandwidth, and computational load will increasingly be treated as dynamic resources that can be sensed and controlled. The round-trip-time modeling in [9], the digital twin infrastructure in [11], and communication-aware neuroadaptive control in [17] all point to this direction. This will be especially important for telerobotics, swarms of mobile robots, remote lab applications (like pharma automation), and medical systems, where decision-making may be distributed across heterogeneous hardware and administrative domains.

Third, digital twins are evolving from visualization tools into executable, continuously updated operational models. Their value will increasingly lie in synchronizing sensing, simulation, planning, diagnosis, and control. The digital twin becomes a bridge between the physical system and its computational representation, enabling safer testing of decisions before these are executed and supporting benchmarking and lifecycle verification after deployment. Such architectures naturally align with the systems-of-systems perspective recently articulated by Kacprzyk, Kovács, and Rudas [5].

Fourth, the boundary between “sensor technology” and “robotics research” will continue to erode. Reliable autonomy depends on procedures for validating, calibrating, and interpreting them under realistic motion and environmental conditions. The sensor-validation approach in [12] is therefore emblematic of a wider trend toward automated metrology and evidence-based deployment. For robots operating near people, the same principle extends to perception confidence, semantic safety assessment, and human-centered interaction [7,13].

Fifth, verification will have to be scaled from components to behavior. Conventional validation often tests sensors, controllers, and software modules separately, whereas autonomous systems fail through interactions among them: a small sensing bias may alter a learned decision, a communication delay may change a control action, or a human response may invalidate an assumed operating condition. Future certification strategies will therefore need layered evidence combining component metrology, scenario-based testing, simulation, digital twins, statistical robustness, and runtime monitoring. The contributions in [7,9,11,12,13] already illustrate several elements of this emerging validation stack.

Sixth, the expected rise of general/multimodal AI in robotics should be viewed as an architectural opportunity. World Models that interpret language, images, sensor streams, and task context may substantially improve task specification and adaptation, particularly in heterogeneous workflows such as those represented through this Special Issue in pathology [8,10] and archeology [15]. Their stochastic and data-dependent behavior reinforces the need for explicit constraints, calibrated sensing, deterministic safety layers, and auditable execution.

The autonomous system of the future will often not be a single robot or a physical AI. It may be a laboratory workflow, a manufacturing cell, a teleoperated medical service, an archeological exploration pipeline, or a coordinated collection of sensors, robots, digital models, and human experts. This interpretation is consistent with the hierarchical view of intelligence and autonomy developed by Wang et al. [4] and with the emerging system-of-systems perspective [5].

## 4. Concluding Perspective

This Special Issue exhibits how the field of intelligent robotics is simultaneously becoming more complex and more demanding. Robotics is not only absorbing powerful learning methods, but it is also rediscovering the importance of structure, uncertainty management, timing, validation, and systems engineering. The resulting convergence is closely aligned with the intellectual legacy of Professor Imre J. Rudas: intelligence as an engineered synthesis of complementary methods rather than a single dominant technique. This collection captures a transition toward integrated autonomy in which sensing, computation, communication, control, and human interaction must be designed together. We hope that these papers will encourage further work on robotic systems that are not only capable, but also trustworthy, adaptive and deployable in the complex environments where intelligent technology can have its greatest impact.
